# Clinical outcome and prognostic factors for patients treated within the context of a phase I study: the Royal Marsden Hospital experience

**DOI:** 10.1038/sj.bjc.6604218

**Published:** 2008-03-18

**Authors:** H-T Arkenau, D Olmos, J E Ang, J de Bono, I Judson, S Kaye

**Affiliations:** 1 Drug Development Unit, Royal Marsden Hospital and Institute of Cancer Research, Downs Road, Sutton SM2 5PT, UK

**Keywords:** phase I trial, outcome, survival, prognostic factors

## Abstract

The main aim of phase I trials is to evaluate the tolerability and pharmacology of a new compound. However, investigating the potential for clinical benefit is also a key objective. Our phase I trial portfolio incorporates a range of new drugs, including molecular targeted agents, sometimes given together with cytotoxic agents. We performed this analysis of response rate, progression-free (PFS) and overall survival (OS) to assess the extent of clinical benefit rate (CBR: partial response (PR)+stable disease (SD)) derived from current trials. We analysed 212 consecutive patients who were treated in 29 phase I studies, from January 2005 to June 2006. All patients had progression of disease prior to study entry. The median age was 58 years (range: 18–86) with a male/female ratio of 2 : 1. A total of 148 patients (70%) were treated in ‘first in human trials’ involving biological agents (132 patients) or new cytotoxic compounds (16 patients) alone, and 64 patients (30%) received chemotherapy-based regimens with or without biological agents. After a median follow-up time of 34 weeks, the median PFS and OS were 11 and 43 weeks, respectively. The CBR was 53% (9% PR and 44% SD) after the first tumour evaluation after two cycles (between weeks 6 and 8) and has been maintained at 36 and 26% at 3 and 6 months, respectively. Treatment related deaths occurred in 0.47% of our patients and treatment had to be withdrawn in 11.8% of patients due to toxicity. A multivariate analysis (MVA) of 13 factors indicated that low albumin (<35 g l^−1^), lactate dehydrogenase>upper normal limit and >2 sites of metastasis were independent negative prognostic factors for OS. A risk score based on the MVA revealed that patients with a score of 2–3 had a significantly shorter OS compared to patients with a score of 0–1 (24.9 weeks, 95% CI 19.5–30.2 *vs* 74.1 weeks, 95% CI 53.2–96.2). This analysis shows that a significant number of patients who develop disease progression while receiving standard therapy derived benefit from participation in phase I trials. Risk scoring based on objective clinical parameters indicated that patients with a high score had a significantly shorter OS, and this may help in the process of patient selection for phase I trial entry.

Patients with advanced cancers most commonly face the dilemma of having no available standard treatment option. A minority of patients with good performance status (PS) and adequate organ function are sometimes offered treatment within the context of a phase I trial. Phase I trials are designed primarily to evaluate the tolerability and toxicity profile of new therapies. The generally accepted inclusion and exclusion criteria for these trials include adequate organ function and reasonable PS in order to ensure safety and avoid unnecessary toxicity. Another important entry criterion is life expectancy predicted to be more than 3 months, and this is notoriously difficult to predict. More accurate selection criteria or even prognostic scores for patients who will potentially benefit from a clinical phase I trial may therefore be helpful.

So far, there have been few studies exploring factors associated with clinical outcome, toxicity and prognosis in this context. Multivariate analyses (MVAs) have revealed that factors such as poor PS, high lactate dehydrogenase (LDH), low albumin, and certain chemotherapy regimens could be negative prognostic factors for survival. However, one of the drawbacks of these studies has been that most analyses have been performed over a long period of time, some of them over 10 years. Moreover, most of these studies focused on the ‘classical cytotoxic drug development era’ and not on the newer generations of molecularly targeted or biological agents (Yamamoto *et al*, 1999; Han *et al*, 2003; Penel *et al*, 2008).

Biological agents target a certain molecular structure or pathway relevant for cancer growth. Broadly speaking, the mechanism of action usually results in a cytostatic rather than cytotoxic effect, resulting in lower toxicity to normal tissue.

We performed this retrospective analysis in all patients who took part in phase I trials at the Drug Development Unit, Royal Marsden Hospital, over an 18-month period, from January 2005 to June 2006. During this period, the majority of our trials involved biological agents. Clinical parameters, blood tests (biochemistry and full blood count (FBC)), tumour type, toxicity and type of treatment were included in our univariate and MVAs. The main aim of this study was to analyse the clinical outcome for our large patient population treated in phase I trials. Secondly, we were interested in the impact of the type of phase I trial on clinical outcome. Our third aim was to analyse factors that could guide us in the development of an improved patient selection process for phase I trials.

## PATIENTS AND METHODS

### Patient characteristics

We analysed the outcome of 212 consecutive patients who were treated from January 2005 to June 2006 in 29 phase I trials at the Drug Development Unit, Royal Marsden Hospital, Sutton, UK. All patients had to have objective evidence of progressive disease prior to trial entry. The median age was 58 years (range: 18–86) with a male/female ratio of 2 : 1 (142 male and 70 female). Overall, the patients had a median of two cycles of prior systemic therapy (range 0–8). The Eastern Cooperative Oncology Group (ECOG) PS was 0, 1 and 2 in 28, 66 and 6% of patients, respectively. A total of 33.5% of the patients had urological tumours, 15.6% had breast and gynaecological tumours, 14.2% had lung, mesothelioma and head and neck tumours, 12.3% had sarcoma, 12.3% had gastrointestinal tumours, 6.1% had melanoma, and 6.1% others. Of the patients with urological tumours, 37 of 54 had prostate cancer previously untreated with chemotherapy. Sixty-four per cent had ⩽2 sites of metastasis and 36% had ⩾2 sites of metastasis (median 2, range: 0–8). The most common sites of metastasis were lung (41%), bone (29%) and liver (27%). Baseline biochemistry showed decreased albumin levels in 57% of the patients (albumin <35 g l^−1^) and LDH levels were above the upper normal limit (UNL: >192 IU) in 49%. The FBC showed haemoglobin levels <12 g dl^−1^ in 41%, a white cell count (WCC) >10 500 mm^−3^ in 11% and platelets >400 000 mm^−3^ in 24% of the patients (Table 1).

### Trial characteristics

During the 18-month study period, all 212 consecutive patients were treated within one of 29 phase I trials. A total of 148 patients (70%) were treated in ‘first in human trials’ involving biological agents (132 patients) or new cytotoxic compounds (16 patients) alone and 64 patients (30%) received chemotherapy-based regimens with or without biological agents. According to the various protocols, the first tumour evaluation was performed before the third cycle, between weeks 6 and 8. Trial categories are summarised in Table 1.

Of our 29 trials, 19 trials have already been completed and phase II doses were recommended; 17 of the 19 trials reached maximum-tolerated doses (MTDs). Three trials were closed early based on the sponsor's decision. Seven trials are still ongoing and four of them have reached MTDs. Overall, MTDs were defined for 21 of the 29 trials.

### Statistical consideration

The SPSS Programme (Version 12.0, Chicago, IL, USA) was used for statistical analysis. The Kaplan–Meier method was used to estimate progression-free (PFS) and overall survival (OS) and the log-rank test was used to compare the survival curves (Kaplan and Meier, 1958). The Cox regression model has been applied for HR estimation and for MVA of prognostic factors for PFS and OS, using a backward selection approach (Cox, 1972). Fisher's exact test and the *χ*^2^ test for trend were used to compare proportions of response rate (RR). All *P*-values presented are two-sided. The cutoff date for the present analysis was the 31 May 2007.

### Outcome: response, PFS and OS

Of the 212 patients, 202 (95%) had disease evaluable by the acknowledged standard Response Evaluation Criteria in Solid Tumours (RECIST) and Prostate Specific Antigen Working Group criteria (PSAWG) were also used to determine progressive disease but not response in this specific setting (Bubley *et al*, 1999; Therasse *et al*, 2000). Overall, there has been a radiological proven partial response (PR) in 19 patients (9.4%), stable disease (SD) in 88 patients (44%) and progression of disease in 95 patients (47%) of all patients, respectively. The clinical benefit rate (CBR: PR+SD) was 53% after the first tumour evaluation after two cycles of treatment (between weeks 6 and 8) and has been maintained at 36 and 26% at 3 and 6 months, respectively. Patients who received a chemotherapy-based regimen had a RR of 19.7% and patients who received a biological agent 3.6% (*P*=0.01). The median duration of treatment was 6.9 weeks (range: 0–60.1 weeks) in patients who received a biological agent and 10.6 weeks (range: 0.1–64.4 weeks, *P*=0.027) in patients who received a chemotherapy-based regimen with or without a biological agent. The 30- and 90-day mortality was 1.9% (4 out of 212) and 18.3% (39 out of 212), respectively. Treatment related mortality was 0.47% (1 out of 212) and 11.8% (25 out of 212) of the patients have been withdrawn from an ongoing study due to toxicity (Table 2).

**Figure 1:**
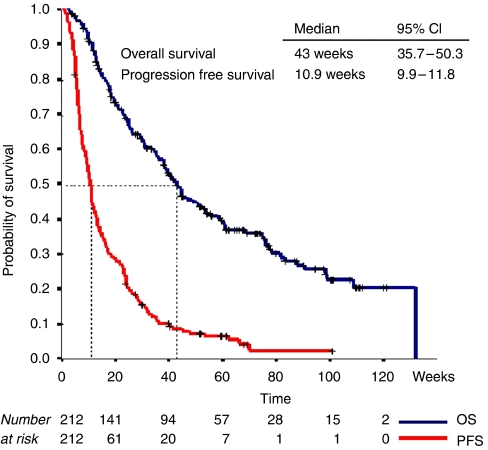
Kaplan–Meier Curves for progression-free (PFS) and overall survival (OS).

After a median follow-up of 34 weeks (range: 2.7–131.9), the PFS was 11 weeks (95% CI: 9.9–11.8) and the median OS 43 weeks (95% CI: 3.8–50.3), respectively (Figure 1). Univariate analysis revealed that ECOG PS 2, >2 sites of metastasis, albumin <35 g day^−1^, LDH>UNL, WCC >10 500 mm^−3^, haemoglobin <12 g dl^−1^, platelets >400 000 mm^−3^, among other factors, were highly significant negative prognosticators for OS. In the MVA, LDH>UNL (*P*=0.003), albumin <35 g dl^−1^ (*P*<0.001), and >2 sites of metastasis (*P*=0.01) were significant negative prognosticators for OS (Table 3). Since our patient population included a number of chemonaive prostate cancer patients and our trial portfolio included docetaxel regimens, we also assessed the outcome according to disease type; urologic *vs* non-urologic cancer. Urologic cancers were associated with a significant better outcome compared to non-urologic cancers, *P*=0.001 (Table 3).

**Figure 2:**
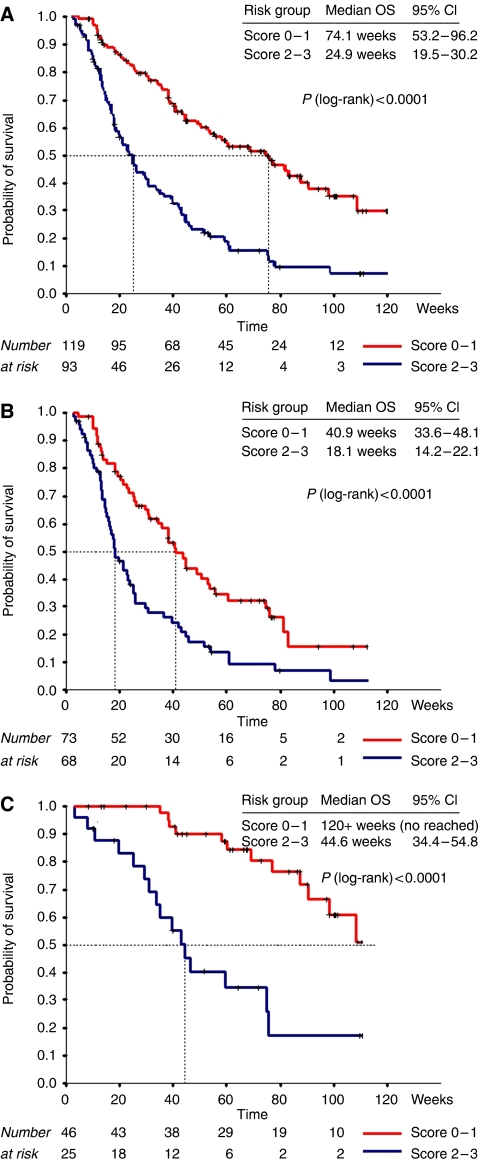
OS by risk categories. Kaplan–Meier curves for OS based on MVA risk score (albumin <35 g l^−1^, +1; elevated LDH>UNL, +1; >2 sites of metastasis, +1). (**A**) Whole series (*n*=212), score 0–1 (*n*=119) and score 2–3 (*n*=93). (**B**) Non-urological cancers (*n*=141), score 0–1 (*n*=73) and score 2–3 (*n*=68). (**C**) Urological tumours (*n*=71), score 0–1 (*n*=46) and score 2–3 (*n*=25).

A risk score based on the results of the outcome of the MVA (LDH normal=0 *vs* LDH>UNL=1, albumin >35 g l^−1^=0 *vs* <35 g l^−1^=1, site of metastasis <2=0 *vs* >2=1) demonstrated that patients with a score <2 had a significantly longer OS (74.1 *vs* 24.9 weeks, *P*=0.0001). Moreover, the same score remained highly predictive for urological patients, a subgroup with a significant better OS, and could also distinguish between a good and poor prognosis cohort in this group of patients (Figure 2A, B and C).

## DISCUSSION

The primary objectives of a phase I trial have classically been to determine the toxicity profile of a new drug therapy and its MTD. However, the clinical outcome measured in RR, PFS and OS is usually descriptive due to the small numbers of patients enrolled in these studies.

Previous retrospective analyses have studied the outcome for patients on phase I trials and have reported RRs between 3.8 and 17.8% with higher RR in patients who received classical cytotoxic drugs compared to patients who received biological agents (Sekine *et al*, 2002; Roberts *et al*, 2004; Horstmann *et al*, 2005). These studies however reviewed the outcome over a long period of time, sometimes more than 10 years, which may not reflect the current status of drug development. Moreover, these trials analysed only the published outcome of clinical trials rather than individual patient data.

In our patient group, with a broad spectrum of different cancers, the RR was 9.4% and median PFS and OS were 11 and 43 weeks, respectively, after a median follow-up of 34 weeks. Patients who received a chemotherapy-based regimen with or without a biological agent had significantly higher RR compared to patients who received non-cytotoxic agents (19.7 *vs* 3.6%). These results are in keeping with the aforementioned published analysis comprising more than 460 phase I trials over a 12-year period, which found an RR of 17.8% for patients who received a chemotherapy-based regimen compared to 4.4% for patients who received non-cytotoxic agents. No data were available on patients achieving SD, nor was a survival analysis performed in that study (Horstmann *et al*, 2005).

A patient-specific analysis was performed in a single centre retrospective analysis, which enrolled 420 patients treated within 16 phase I trials over a 10-year period. This study showed OS rates of 38 weeks for patients who received cytotoxic-based regimens compared to 27 weeks for patients who received non-cytotoxic treatment. These results were similar to our survival analysis, which showed that classical cytotoxic-based regimens resulted in longer OS compared to biological agents. This trial also confirmed that RR was significantly higher in patients receiving cytotoxic agents compared to patients who received non-cytotoxic agents (14.1 *vs* 1%, *P*<0.001) (Han *et al*, 2003).

Our series demonstrated a better outcome for patients who had disease control (CBR: PR+SD), which was reflected in an overall CBR of 36% at 3 months and 26% at 6 months, respectively. It is notoriously difficult to attribute a better outcome to treatment effect in a non-randomised study analysis where patient selection clearly is a major factor. However, the achievement of SD lasting more than 3 months in advanced cancer patients with previous disease progression is noteworthy, and in several cases has justified the further development of the agent under investigation.

The patients who died within the first 3 months of treatment reflected a group with an unfavourable prognosis. Our analysis showed that these patients had significantly higher LDH, WCC and lower albumin and haemoglobin levels compared to the rest of the population. Similar results were demonstrated in a study, which included 70 phase I patients. All patients who presented with the following two risk factors, albumin <38 g l^−1^ and lymphocyte count <0.7 × 10^9^ l^−1^, died within 90 days (Penel *et al*, 2008). Another study, which analysed 154 patients over an 8-year period, identified two independent risk factors, namely LDH >600 IU and PS >1, which were correlated with a shorter 90-day OS for patients with both factors. The authors recommended that patients with these risk factors should not participate in a phase I trial (Bachelot *et al*, 2000). Interestingly, this study also revealed that patients ⩾65 years had a significantly better OS than younger patients in keeping with our findings. A possible explanation includes more aggressive tumour biology in younger patients.

In our MVA, parameters such as LDH>UNL, albumin <35 g l^−1^, sites of metastasis >2 have been associated with a significantly poorer clinical outcome. On the basis of these results, a prognostic score model was developed (LDH normal (0) *vs* LDH>UNL (+1), albumin >35 g l^−1^ (0) *vs* albumin <35 g l^−1^ (+1), site of metastasis <2 (0) *vs* >2 (+1)). Our prognostic score demonstrated that patient with a good risk score (0 and 1 risk factors) had significantly superior OS compared to patients with a poor risk score (>2 risk factors). This score has been also proved to be valid in the subgroup of urological cancer patients. The use of this score might be helpful for the future as it is based solely on objective clinical parameters. It could be a helpful tool in evaluating the eligibility of patients into phase I trials. We are currently performing a prospective analysis in our phase I patients to validate this scoring system.

This analysis demonstrated that treatment within the context of a phase I trial could be considered as a valuable therapeutic option. Interestingly, those trials incorporating classical cytotoxics were associated with a better outcome. Clearly, this relates to patient selection, particularly when the trial may involve the use of a cytotoxic in chemonaive cases. The treatment in our cohort was generally well tolerated and treatment-related deaths and toxicities were low. Moreover, a significant number of patients achieved disease control for a significant duration. However, the challenge remains in appropriate patient selection and for this, the use of an objective clinical score could be a helpful tool.

## Figures and Tables

**Table 1:** Baseline patient and treatment characteristics

**Characteristics**	**Median (range)**	**Number**	**%**
*Sex*			
Male		142	67
Female		70	33
			
*Age*	58 years (19–86)		
<65 years		147	69
⩾65 years		65	31
			
*Performance status*		208	
ECOG 0		58	28
ECOG 1		137	66
ECOG 2		13	6
			
*Previous treatments*	2 (0–8)		
0–2 previous systemic lines		110	52
⩾3 previous systemic lines		102	48
			
*Number of involved areas*	2 (0–8)		
Only locoregional disease		14	7
1–2 metastatic sites/areas		121	57
⩾3 metastatic sites/areas		77	36
			
*Metastatic specific sites*			
Liver		57	27
Lung		86	41
Bone		62	29
			
*Baseline albumin*	33 g l^−1^ (18–44)		
<35 g l^−1^		91	57
⩾35 g l^−1^		121	43
			
*Baseline LDH level*	190 IU dl^−1^ (55–2024)		
Normal LDH		108	51
Elevated LDH		104	49
			
*Baseline Haemoglobin level*	11.95 g dl^−1^ (8.7–16.0)		
<12 g dl^−1^		86	41
⩾12 g dl^−1^		126	59
			
*Baseline WBC count*	7150 mm^−3^ (2900–21200)		
⩽10 500 mm^−3^		188	89
>10 500 mm^−3^		24	11
			
*Baseline platelets count*	284000 mm^−3^ (108000–797000)		
⩽400 000 mm^−3^		162	76
>400 000 mm^−3^		50	24
			
*Cancer group*		208	
Urological tumours		71	34
Breast and gynaecological cancers		33	16
Gastrointestinal cancers		26	12
Sarcomas		26	12
Thoracic and head and neck tumours		30	14
Melanoma		13	6
Others		13	6
			
*Trial categories*			
‘First in human drugs’		148	70
Cytotoxic drug combinations (including FDA approved drugs)		64	30
			
*Trial categories by target*			
Growth factor receptor pathways		63	30
Chromatin remodelling, DNA repair and antisense		41	19
Anti-angiogenesis		38	18
Cell cycle and apoptosis		27	13
Vaccine and virus		16	7.5
New cytotoxic compounds		16	7.5
Hormone synthesis		8	4
Protein turnover		3	1

**Table 2:** Trial responses and outcomes

		**‘First in human’**		
	**Overall**	**Median (range) – n/N (%)**	**Chemotherapy-based**	***P*-value**
Number of Cycles	2 (1–17)	2 (1–17)	4 (1–17)	**<0.001**
Treatment (weeks)	7.7	6.9	10.6	**0.027**
Partial response	19/202 (9.4)	5/140 (3.6)	14/62 (22.6)	**0.001**
Stable disease >3 months	54/202 (26.7)	31/140 (22.1)	23/62 (37.1)	**<0.01**
CBR3 m (PR+SD>3 months)	73/202 (36.1)	36/140 (25.7)	37/62 (59.7)	**0.001**
30 days mortality rate	4/212 (1.9)	2/148 (1.3)	2/64 (3.1)	NS
90 days mortality rate	39/212 (18.3)	28/148 (18.9)	11/64 (17.2)	NS
Toxicity-related mortality	1/212 (0.47)	1/148 (0.7)	0/64 (0)	NS
Off-trial due to toxicity	25/212 (11.8)	19/148 (12.8)	6/64 (9.4)	NS

CBR_3m_=3 months clinical benefit rate; NS=not significant; PR=partial response; SD=stable disease.

*P*-values calculated by Mann–Withney's *U*-test, *χ*^2^ test and Fisher's *F*-test.

**Table 3:** Overall survival and prognostic factor categories (log-rank test for univariate analysis and Cox regression for multivariate analysis)

			**Statistical analysis**	
**(*n*=212)**	**Median (weeks)**	**95% CI**	**Univariate Log Rank**	**Multivariate Cox Reg**
Albumin <35 g l^−1^	26	18.6–33.4	**<0.0001**	**0.007**
Normal albumin	74	54.3–95.1		
Elevated LDH	34	24.3–44.0	**0.003**	**0.002**
Normal LDH	59	41.6–77.0		
WCC >10 500 mm^−3^	16	5.7–26.3	<0.0001	0.439
Normal WCC	47	36.6–56.8		
HGB <12 g dl^−1^	31	22.4–39.0	0.0001	0.309
Normal HGB	60	37.9–82.4		
PLT >400 000 mm^−3^	23	8.3–37.7	0.0035	0.394
Normal PLT	47	35.8–57.4		
>2 MTS sites	30	18.3–40.8	**0.0007**	**0.025**
0–2 MTS sites	52	38.1–65.0		
Female	26	11.6–40.4	0.027	0.618
Male	54	39.7–67.5		
<65 years	38	30.1–46.2	0.0271	0.343
⩾65 years	60	25.6–94.6		
Liver MTS	25	22.7–27.0	0.0186	0.574
No liver MTS	47	36.8–56.6		
Lung MTS	36	25.3–45.6	0.0234	0.0804
No Lung MTS	54	38.5–45.8		
No Bone MTS	38	29.8–46.4	0.0004	0.425
Bone MTS	88	60.1–115		
Non-urologic tumours	26	17.8–34.8	**<0.0001**	**0.001**
Urologic tumours	98	67.6–129		
ECOG 0–1	44	34.8–54.3	0.0048	0.376
ECOG 2	17	12.6–22.8		
Monotherapy trial	38	44.9–108	0.0005	0.157
Combination trial	76	28.4–47.8		
